# Molecular mechanism of tumor-infiltrating immune cells regulating endometrial carcinoma

**DOI:** 10.1016/j.gendis.2024.101442

**Published:** 2024-11-05

**Authors:** Silu Ding, Yingying Hao, Yue Qi, Heng Wei, Jin Zhang, Hui Li

**Affiliations:** a Department of Radiation Oncology, The First Hospital of China Medical University, Shenyang, Liaoning 117004, China; b Department of Obstetrics and Gynecology, Shengjing Hospital of China Medical University, Shenyang, Liaoning 117004, China; c Department of Gynecology, The First Hospital of China Medical University, Shenyang, Liaoning 117004, China

**Keywords:** Clinical implication, Endometrial carcinoma, Immune checkpoint pathways, Immune evasion strategies, Tumor-infiltrating immune cells

## Abstract

Endometrial carcinoma (EC) is a prevalent gynecological cancer, and its interaction with the immune system is pivotal in cancer progression. This comprehensive review explores the molecular mechanisms involved in the regulation of EC by tumor-infiltrating immune cells. This review discusses the composition and functions of various immune cell types within the tumor microenvironment, including T cells, B cells, macrophages, and natural killer cells, and elucidates their specific roles in cancer control. It also delves into the immune evasion strategies employed by EC cells, with a specific focus on immune checkpoint pathways and their influence on tumor development. Signaling pathways, cytokines, and chemokines mediating immune responses within the tumor microenvironment are also detailed. Furthermore, clinical implications and therapeutic strategies, such as immunotherapies, are also reviewed, and relevant clinical trials are discussed. Additionally, this review discusses the existing gaps in our knowledge, suggests potential avenues for future research, and emphasizes the significance of understanding these mechanisms for enhanced EC treatment. This review provides an exhaustive overview of the current knowledge, supporting the ongoing quest for more effective therapeutic interventions on EC.

## Introduction

Endometrial carcinoma (EC), a prevalent gynecological cancer, develops within the uterine lining, the endometrium. Given its rising incidence and significant impact on women’s health, the multifaceted nature of EC has received substantial attention in oncology.^1^, ^2^, ^3^ This cancer predominantly affects postmenopausal women, although occurrences in younger age groups, often associated with obesity and hormonal imbalances like polycystic ovary syndrome, are not uncommon. The prevalence of EC varies globally, with higher rates in North America and Europe attributed to lifestyle factors and increased life expectancy.^4^^,^^5^ EC is also on the rise in Asian regions such as China and Japan.^6^^,^^7^ Typically, EC falls into two histological subtypes. Type I EC is estrogen-dependent and its development may occur under the long-term influence of estrogen without progesterone antagonism, resulting in endometrial hyperplasia, atypical hyperplasia, and subsequently, carcinogenesis. Type II EC is estrogen-independent, and its onset is not clearly associated with estrogen levels. This category of EC includes less common pathological types, such as endometrial serous carcinoma, clear cell carcinoma, and carcinosarcoma.^8^^,^^9^ While type I EC is more frequent and generally associated with a better prognosis, type II EC is more aggressive and is frequently diagnosed in its advanced stage.^10^^,^^11^ In 2013, The Cancer Genome Atlas project gave four new classifications for EC,^12^ including DNA polymerase epsilon mutated subtype, mismatch repair deficient subtype, no specific molecular subtype, and p53 abnormal subtype. The progression of EC, irrespective of the dynamic changes in the tumor microenvironment (TME), can be likened to the solution of the Sphinx’s riddle. Comprehending the molecular mechanisms underlying the development and progression of these subtypes is crucial for developing tailored therapies and personalized treatment strategies.

The TME, enriched with tumor-infiltrating immune cells such as T cells, B cells, macrophages, natural killer (NK) cells, and dendritic cells, acts as a double-edged sword in EC. This dynamic environment, where immune cells can either suppress or promote cancer growth, is crucial for dictating disease progression and significantly impacts therapeutic outcomes.^13^, ^14^, ^15^, ^16^ This dichotomous function of the immune system, capable of both supporting and inhibiting tumor development, is crucial to understanding and developing effective immunotherapies. Advances in our knowledge of the molecular interactions between tumors and immune cells are essential for refining treatment strategies and harnessing the natural defense mechanisms of the immune system against cancer.^13^^,^^17^

**Figure 1:**
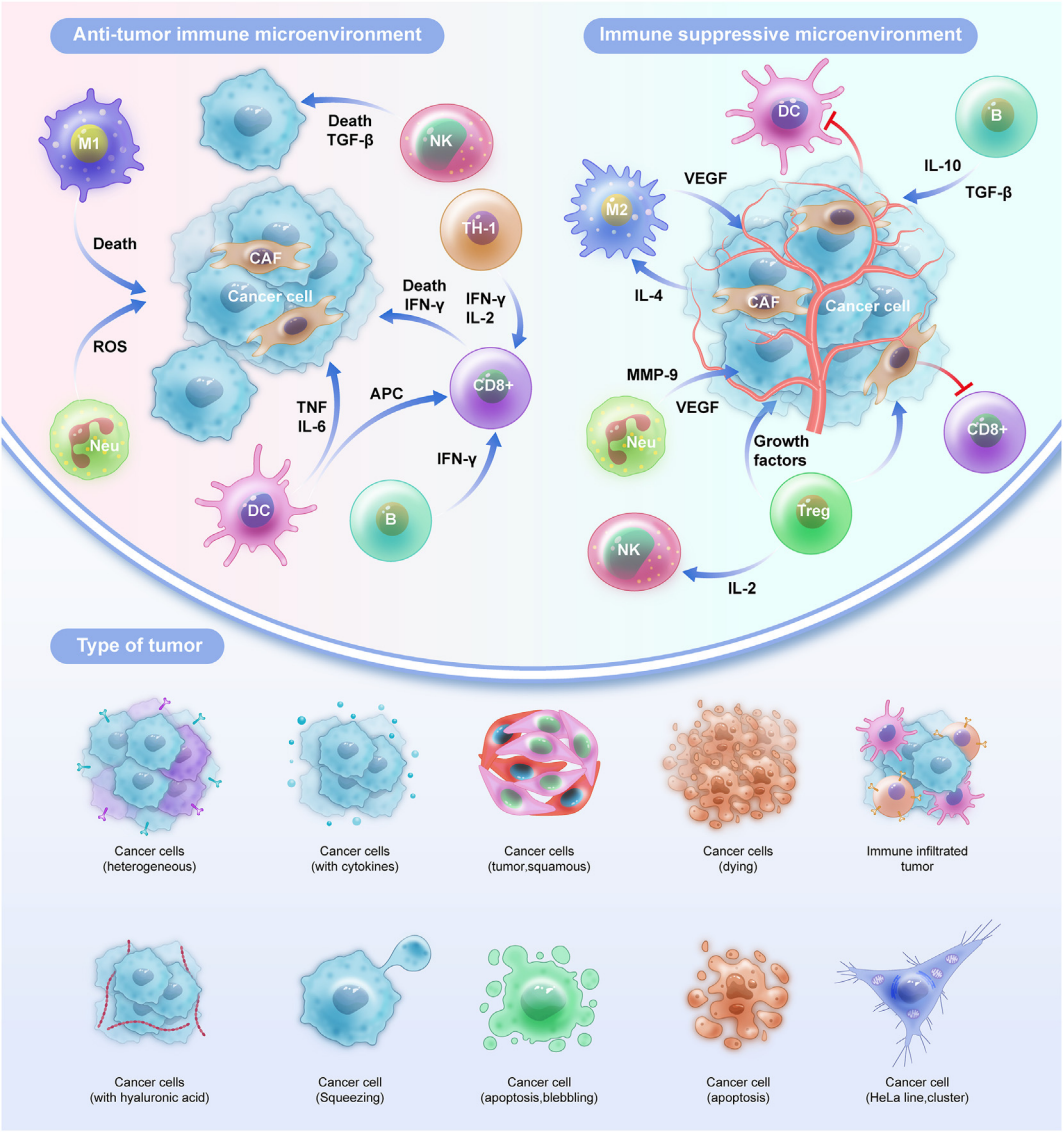
Endometrial carcinoma involves an intricate interplay between tumor-infiltrating immune cells, comprising a multifaceted microenvironment where opposing forces can either enhance or suppress tumor progression. This dichotomy arises from the dynamic equilibrium maintained by immune cells within the tumor microenvironment. While certain immune components exert anti-tumor effects, a cadre of cells specializing in immune evasion fosters tumor growth. This complex immune environment highlights the need to understand the detailed molecular mechanisms at play, so we can better use this knowledge for effective treatments and personalized strategies.

EC exhibits complex interplays between tumor-infiltrating immune cells that can promote or inhibit tumor growth^18^, ^19^, ^20^ (Fig. 1). This duality arises from the complex and dynamic nature of the immune response to cancer. Immune cells can recognize and eliminate cancer cells through immunosurveillance, however, the TME can induce immune suppression, enabling cancer cells to evade detection and proliferate unchecked. These intricate interactions often involve immune checkpoint pathways such as programmed cell death protein 1/programmed cell death ligand 1 (PD-1/PD-L1), creating a dynamic battleground in which cancer and the immune system engage in an ongoing struggle.^16^^,^^21^

With the multifaceted objectives of this comprehensive review, we aim to provide a comprehensive understanding of the immune microenvironment in EC and elucidate the composition and functions of tumor-infiltrating immune cells. Next, we delve into the specific molecular mechanisms these immune cells use to promote or restrain cancer progression. To this end, we thoroughly explore various immune cell types, their respective roles, and their contributions to the TME. Third, the strategies employed by EC cells to evade immune recognition are explored, focusing on immune checkpoint pathways and their implications. We also dissect the signaling pathways and cytokines that mediate immune responses within the TME, shedding light on the intricate cross-talks between immune cells and cancer cells. We finally discuss the clinical implications of understanding these molecular mechanisms, with a focus on the significance of ongoing research in this field for the development of more effective treatment strategies for EC. Therefore, this review seeks to contribute to the expanding body of knowledge concerning the immune regulation of EC, providing insights for future research and clinical practice.

## Immune microenvironment in EC

### Composition of tumor-infiltrating immune cells

The composition of tumor-infiltrating immune cells in the context of EC presents a multifaceted and dynamic assemblage of distinct cell types that shape the intricate TME.^13^^,^^22^^,^^23^ This intricate cellular landscape encompasses a variety of immune cells, each with unique functions that can significantly impact cancer progression. In patients with endometrial subtypes with high microsatellite instability, the response to immunotherapy was associated with a higher rate of tumor-infiltrating immune cells. Furthermore, the authors concluded that the abundance of tumor-infiltrating immune cells is an independent prognostic factor with better accuracy than the high microsatellite instability status.^24^

T lymphocytes are prominently featured in the immune infiltration of EC, which encompass both CD4^+^ (helper) and CD8^+^ (cytotoxic) T cells. CD8^+^ T cells hold a pivotal role in recognizing and eradicating cancer cells, while CD4^+^ T cells orchestrate the immune response by activating other immune cells and cytokine production.^15^^,^^25^^,^^26^ In a less abundant capacity, B cells have been identified within the TME, possessing the ability to produce antibodies and cytokines, thereby participating in immune response regulation against EC.^17^ Tumor-associated macrophages represent a substantial portion of the immune infiltrate and exhibit divergent roles contingent upon their polarization.^27^^,^^28^ They are classified as M1 (anti-tumor, pro-inflammatory) or M2 (pro-tumor, anti-inflammatory) macrophages, and their balance significantly influences the tumor behavior. Another cell constituting innate immune cells, the NK cells, play a critical role in tumor surveillance and cytotoxicity^29^^,^^30^ by recognizing and eliminating cancer cells within EC. Dendritic cells, as antigen-presenting cells, initiate adaptive immune responses by presenting tumor antigens to T cells, and thus play a crucial role in antigen recognition and the activation of T-cell responses.

Myeloid-derived suppressor cells are a heterogeneous group of cells that exert immunosuppressive functions and can accumulate in the TME, inhibiting anti-tumor immune responses.^31^, ^32^, ^33^ Comprehending the immune cell composition is a pivotal step in elucidating the intricate dynamics within the EC microenvironment. Ratios, localization, and interactions between these immune cell types profoundly impact disease progression and response to treatment. Investigations into the specific roles of these immune cells and the governing mechanisms within the context of EC will provide invaluable insights for the development of targeted therapies and immunotherapeutic approaches.

### Role of the immune microenvironment in EC progression

**Figure 2:**
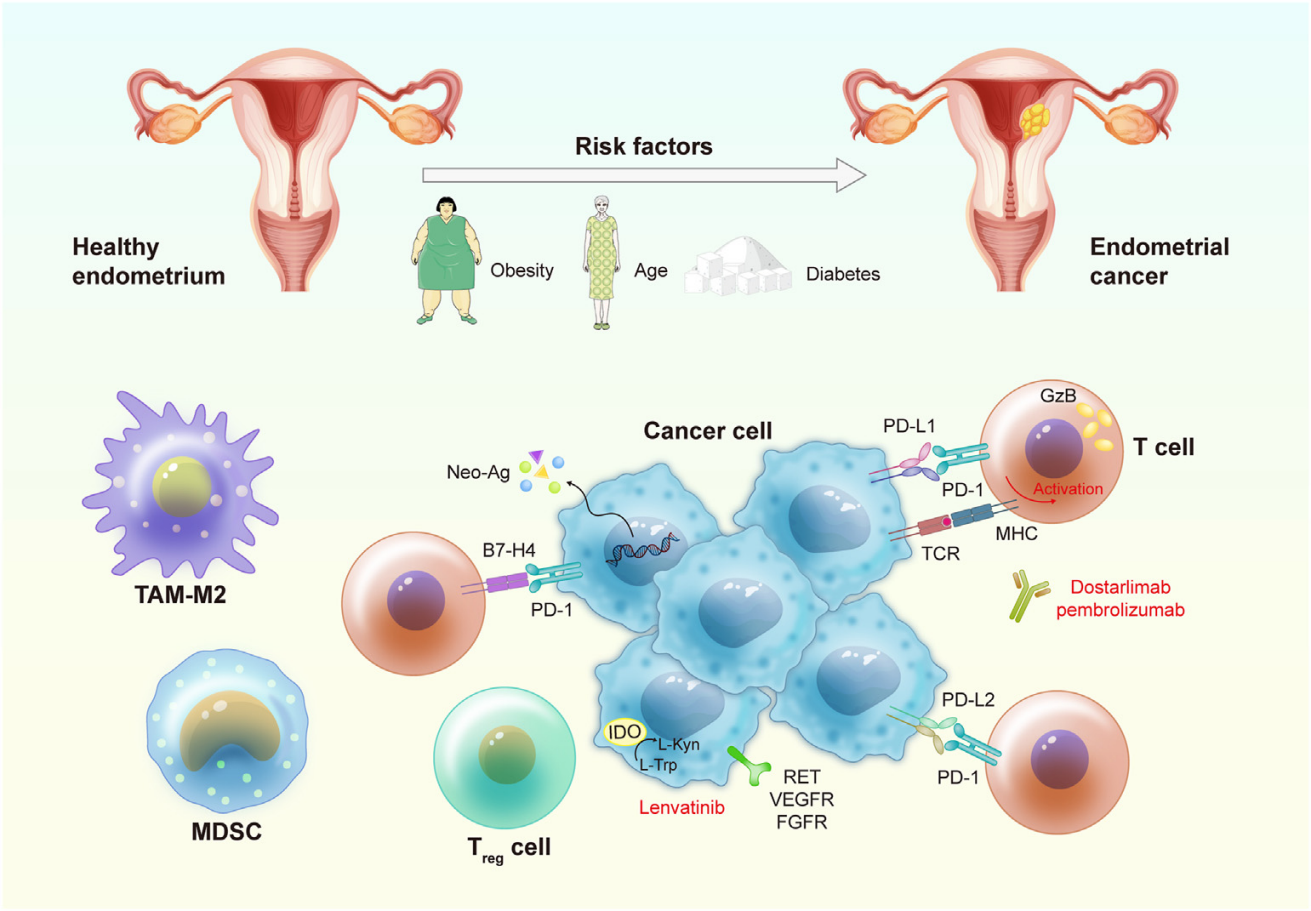
Endometrial carcinoma (EC) development, immune microenvironment, and approved treatments. Risk factors associated with the development of EC include obesity, type 2 diabetes, prior breast or ovarian cancer, and age. The DNA polymerase epsilon (POLE)mut and the microsatellite instability-hypermutated (MSI-H) subtypes of EC have a high mutational load resulting in the production of tumor-specific neo-antigen, favoring cytotoxic T cell infiltration within tumors. EC cells express indoleamine 2,3-dioxygenase (IDO) that converts l-tryptophan (L-Trp) into l-kynurenine (L-Kyn), driving the differentiation of regulatory T cells and inhibiting CD8^+^ and natural killer (NK) cells. EC cells express high levels of rearranged during transfection (RET), vascular endothelial growth factor receptor (VEGFR), and fibroblast growth factor receptor (FGFR), all of which can be targeted with lenvatinib. EC cells also express programmed death-ligand 1 (PD-L1), and the use of dostarlimab or pembrolizumab can facilitate the reactivation of the immune system and the anti-tumor response.

The immune microenvironment of the EC embodies a complex and multifaceted ecosystem that profoundly influences disease progression^13^^,^^34^ (Fig. 2). This dynamic interplay of tumor-infiltrating immune cells with cancer cells assumes a central role in shaping the trajectory of this cancer. Understanding the contribution of the immune microenvironment to cancer progression represents a critical facet of contemporary oncological research. The immune microenvironment, within the context of EC, encompasses various immune cell populations, stromal cells, and an array of cytokines, chemokines, and signaling molecules.^35^^,^^36^ These immune cells play a dual role, serving as both sentinels capable of recognizing and eliminating cancer cells, such as cytotoxic T cells and NK cells, thereby stalling tumor growth, and as contributors to an immunosuppressive environment when modulated by the tumor, promoting tumor progression.

Central to this dual role of immune cells is the immune checkpoint pathway, involving molecules such as PD-1 and PD-L1.^13^^,^^21^^,^^37^ Activation of these pathways can lead to immune evasion, permitting cancer cells to elude detection and destruction. In the context of EC, the expression of these immune checkpoint molecules within the TME emerges as a critical determinant of the tumor’s aggressiveness.^38^, ^39^, ^40^ The immune microenvironment is closely involved in processes including angiogenesis, tissue remodeling, and the establishment of an immunosuppressive niche, immune cell-mediated activities, fibroblasts, and the extracellular matrix. These elements collectively contribute to tumor growth and metastasis. The production of various cytokines and chemokines within the immune microenvironment further influences cancer cell proliferation and invasion and the recruitment of immune cells.

## Immune cell types and their functions

### Diverse immune cell types in the EC microenvironment

T lymphocytes, constituting a central component of the immune infiltrate in EC, exhibit multifaceted roles.^17^^,^^26^^,^^41^ Among T lymphocytes, CD4^+^ T cells and CD8^+^ T cells constitute the highest proportion. B cells, although less prevalent, have been identified within the EC microenvironment^42^^,^^43^; they engage in antibody and cytokine production, potentially influencing immune responses against cancer. The precise role of B cells in EC is being actively investigated. Tumor-associated macrophages represent a significant portion of the immune infiltrate in EC.^44^^,^^45^ They are highly adaptable and can adopt either M1 or M2 phenotypes. M1 macrophages foster an anti-tumor, pro-inflammatory environment, while M2 macrophages display pro-tumor, anti-inflammatory characteristics. The equilibrium between these phenotypes significantly influences carcinoma behavior. In addition, NK cells play a crucial role in identifying and eliminating cancer cells, including those in EC.^30^^,^^46^^,^^47^ Through their cytotoxic function, they target and destroy malignant cells without prior sensitization. Dendritic cells initiate adaptive immune responses by serving as antigen-presenting cells and capturing and presenting tumor antigens to T cells. This process is indispensable for activating effective responses against tumors. Myeloid-derived suppressor cells, known for their immunosuppressive functions, may accumulate in the TME, inhibiting anti-tumor immune responses and fostering an immunosuppressive environment.^31^

### Functions and activities of tumor-infiltrating immune cells in EC

The immune cells infiltrating the cancer microenvironment in EC perform diverse and pivotal roles in regulating cancer progression.^48^^,^^49^ These immune cells collectively affect the equilibrium between anti-tumor immune responses and pro-tumor immune suppression. Summarizing the functions and activities of these immune cells is crucial for understanding their influence on the disease.

T lymphocytes play a central role in the immune response against EC. CD8^+^ cytotoxic T cells are the primary effectors in recognizing and eliminating cancer cells.^41^^,^^50^^,^^51^ Their activity hinges on recognizing tumor-specific antigens, leading to the destruction of malignant cells. CD4^+^ T cells, as also called helper T cells, contribute to immune responses by regulating other immune cells and releasing cytokines. These cytokines modulate the microenvironment, influencing the activation of other immune cells and supporting immune-mediated tumor control. While their role in EC is still an area of active investigation, B cells can produce antibodies and cytokines. These antibodies may bind to cancer cells and mark them for immune recognition and destruction. Additionally, B cells can influence immune responses through cytokine production and interactions with other immune cells. Tumor-associated macrophages are highly plastic cells that can adopt distinct phenotypes. M1 macrophages are pro-inflammatory and anti-tumor, actively participating in immune responses by engulfing and destroying cancer cells.^52^^,^^53^ In contrast, M2 macrophages exhibit anti-inflammatory and pro-tumor characteristics, promoting immunosuppression and tissue remodeling.^54^^,^^55^ NK cells are critical for the early immune response against EC. They can recognize and destroy cancer cells without the need for prior sensitization. NK cells contribute to immune surveillance by identifying and eliminating malignant cells through cytotoxic activity. Dendritic cells are professional antigen-presenting cells. They capture tumor antigens and present them to T cells, initiating adaptive immune responses. Their role is essential in coordinating and enhancing anti-tumor T cell responses. Myeloid-derived suppressor cells exert immunosuppressive effects via various mechanisms, including producing immunosuppressive cytokines (*e.g*., interleukin (IL)-10 and transforming growth factor-beta (TGF-β)) and inhibiting T cell proliferation through nitric oxide and arginase-1.^56^

The functions and activities of these immune cells in EC are closely intertwined and dynamically regulated. A delicate balance exists between immune cells that promote tumor elimination and those that foster tumor progression, making the composition and behavior of the immune microenvironment a key determinant of disease outcome. Understanding these complex interactions is essential for developing targeted therapies that tip the balance in favor of anti-tumor immunity in the fight against EC.

### Specific molecular mechanisms employed by tumor-infiltrating immune cells in EC regulation

Tumor-infiltrating immune cells in the context of EC deploy specific molecular mechanisms to influence disease progression.^18^^,^^48^ Understanding these mechanisms is pivotal for advancing our knowledge of cancer immunology and developing targeted therapeutic strategies. Below, we provide examples of specific molecular mechanisms utilized by key immune cell types, along with references to key studies.

T cells are central to anti-tumor immunity, and their function in EC hinges on antigen recognition and cytotoxic activity. T-cell receptors bind to tumor-specific antigens presented by major histocompatibility complexes on cancer cells.^57^^,^^58^ Once activated, T cells release perforin and granzymes, inducing apoptosis in target cells. Key studies highlight the efficacy of immune checkpoint inhibitors in restoring T cell function in EC.^59^, ^60^, ^61^ While less understood in the context of EC, B cells may contribute to anti-tumor immunity by producing antibodies targeting tumor antigens. This process, known as antibody-dependent cell-mediated cytotoxicity, can enhance the killing of cancer cells. Relevant studies demonstrate the presence of B cell-rich tertiary lymphoid structures in the TME.^62^^,^^63^ Macrophages exhibit distinct phenotypes, with M1 macrophages exerting anti-tumor effects and M2 macrophages promoting immunosuppression. Key mechanisms include the production of pro-inflammatory cytokines (*e.g.*, tumor necrosis factor-alpha and IL-1β) by M1 macrophages and the secretion of immunosuppressive factors (*e.g.*, TGF-β) by M2 macrophages.^64^, ^65^, ^66^ The study emphasizes the impact of macrophage polarization on tumor cell response.^67^ NK cells are pivotal in the early immune response against EC. They employ various receptors, such as NKG2D (natural killer group 2, member D), to recognize stress-induced ligands on cancer cells.^68^ Once engaged, NK cells release cytotoxic granules containing perforin and granzymes, leading to the death of target cells. Dendritic cells capture and present tumor antigens to T cells, initiating adaptive immune responses. The expression of co-stimulatory molecules (*e.g.*, CD80 and CD86) on dendritic cells is essential for T cell activation.^69^ Myeloid-derived suppressor cells exert immunosuppressive effects via various mechanisms, including the production of immunosuppressive cytokines (*e.g.*, IL-10 and TGF-β) and the inhibition of T cell proliferation through nitric oxide and arginase-1.^31^ These examples provide insight into the intricate molecular mechanisms at play in regulating EC by tumor-infiltrating immune cells. A comprehensive understanding of these mechanisms is essential for devising targeted immunotherapies and advancing the treatment of this gynecological malignancy.

## Immune evasion mechanisms by tumor cells

### Evasion of the immune system by EC cells

EC cells employ various strategies to circumvent immune surveillance, significantly contributing to tumor progression and resistance to immunotherapies. An exhaustive comprehension of these evasion mechanisms is imperative for developing efficacious treatment modalities.

**Figure 3:**
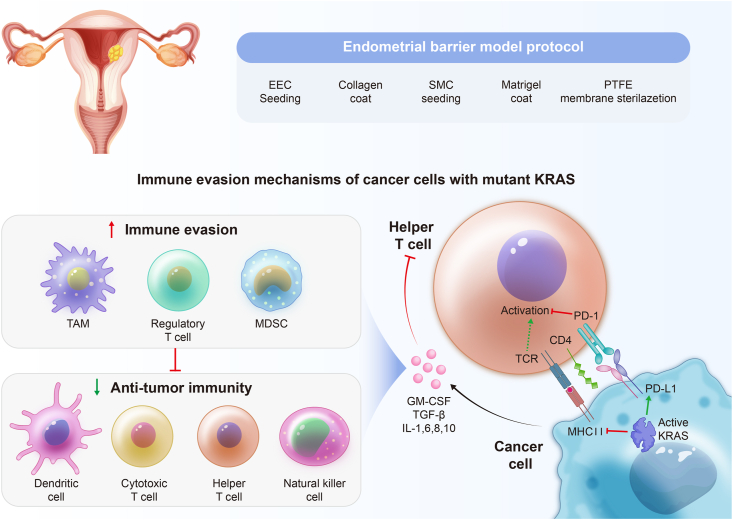
Endometrial carcinoma (EC) cells strategically employ immune checkpoint pathways, with a notable focus on the programmed death-1 (PD-1)/programmed death-ligand 1 (PD-L1) axis. The intricate mechanism, as illustrated, involves the up-regulation of PD-L1 expression by cancer cells, allowing them to interact with PD-1 on T cells. This interaction leads to T cell exhaustion and anergy, effectively suppressing the anti-tumor immune response. Extensive studies have recognized the clinical relevance of these pathways and emphasized their significance in gynecological cancers. Unraveling these complex interactions provides crucial insights for developing immunotherapies tailored to combat EC effectively.

In their quest for immune evasion, EC cells often exploit immune checkpoint pathways, notably PD-1 and its ligand PD-L1^70^^,^^71^ (Fig. 3). Through the up-regulation of PD-L1 expression, cancer cells effectively engage PD-1 on T cells, resulting in T cell exhaustion and anergy.^72^ This interaction adeptly suppresses the anti-tumor immune response. Pertinent studies underscore the clinical relevance of these pathways in the realm of gynecological cancers.^73^^,^^74^ Additionally, EC cells may release immunosuppressive cytokines, including TGF-β and IL-10.^75^^,^^76^ These cytokines play an instrumental role in inhibiting immune cell activation and effector functions, thereby cultivating an immunosuppressive microenvironment that undermines anti-tumor immunity. Furthermore, certain EC cells have evolved mechanisms to impede immune cell ingress into the TME, thereby restricting immune cell access to cancer cells. This exclusion can transpire through modifications in tumor vasculature and stromal elements. Moreover, EC cells may diminish the expression of tumor-specific antigens that are typically recognized by immune cells, thus lowering the likelihood of immune detection and subsequent attack. Lastly, EC can attract immunosuppressive cells, including myeloid-derived suppressor cells and regulatory T cells, into the TME.^25^

A profound grasp of these immune evasion mechanisms is indispensable for formulating targeted therapies capable of surmounting these impediments and augmenting the efficacy of immunotherapies in the battle against EC. Contemporary research endeavors are focused on identifying strategies to reverse or bypass these evasion mechanisms, ultimately enhancing the potency of immunotherapeutic interventions in the combat against this gynecological malignancy.

### Immune checkpoint pathways and their role in immune evasion in EC

Immune checkpoint pathways, exemplified by PD-1 and its ligand, PD-L1, hold a central position in orchestrating immune responses in the milieu of EC. These pathways assume a pivotal role in immune evasion orchestrated by cancer cells and are substantial contributors to the advancement of the tumor. A comprehensive comprehension of the nuances of immune checkpoint signaling is imperative for formulating precisely targeted therapeutic modalities, with the goal of enhancing patient outcomes.

PD-1, expressed on the surface of activated T cells, functions as an inhibitory receptor, and performs an indispensable role in averting excessive immune responses, thereby preserving self-tolerance, and mitigating the risk of autoimmunity.^16^^,^^77^ In contrast, PD-L1 is expressed in various cell types, including cancer cells. When PD-L1 on cancer cells binds to PD-1 on T cells, it suppresses T cell activation and effector functions, compromising the immune system’s ability to combat cancer cells effectively. In EC, cancer cells adeptly exploit the PD-1/PD-L1 pathway to evade immune surveillance. This interaction inhibits the cytotoxic capabilities of T cells, allowing cancer cells to evade immune detection and elimination. Consequently, T cell exhaustion, anergy, and reduced cytokine production collectively suppress the anti-tumor immune response.

Numerous clinical trials have substantiated the potential of immune checkpoint inhibitors, which interdict the interaction between PD-1 and PD-L1, as promising therapeutic strategies with reference to EC.^13^^,^^37^ These inhibitors release the restraints on T cell activity, reinvigorating the immune system’s potential to identify and eradicate cancer cells.

In summation, immune checkpoint pathways, specifically the PD-1/PD-L1 axis, occupy a pivotal role in the immune evasion strategies executed by EC cells. Targeting these pathways through checkpoint inhibitors is a promising approach to fortify anti-tumor immunity and enhance treatment outcomes in this gynecological malignancy.

## Exploring molecular mechanisms of immune evasion in EC

The molecular underpinnings of immune evasion in EC have emerged as a focal point in cancer immunology. The apprehension of how cancer cells undermine immune responses assumes paramount significance in the quest for efficacious therapeutic strategies. Diverse studies have delved into these mechanisms, unveiling the intricacies of immune evasion in the context of EC.

A prominent mechanism of immune evasion in EC revolves around the up-regulation of immune checkpoint molecules, with a particular emphasis on PD-L1 on the surfaces of cancer cells.^78^, ^79^, ^80^ The study has underscored the association between high PD-L1 expression in EC and an unfavorable prognosis, elucidating the significance of this checkpoint molecule in the realm of immune evasion.^80^^,^^81^ Equally noteworthy is the work in treating patients with mismatch repair-deficient EC, providing compelling evidence regarding the clinical relevance of PD-1/PD-L1 interactions.^82^^,^^83^ The secretion of immunosuppressive cytokines by tumor cells is another facet of immune evasion. Among these, TGF-β emerges as a pivotal immunosuppressive cytokine that can be overexpressed by EC cells, culminating in immune suppression.^84^, ^85^, ^86^ Furthermore, the recruitment of regulatory T cells and myeloid-derived suppressor cells into the TME can exacerbate immune evasion.

In conclusion, the molecular mechanisms of immune evasion in EC are multifaceted and dynamic. The studies investigating these mechanisms provide valuable insights into the interactions between cancer cells and the immune system. This knowledge is pivotal for developing targeted immunotherapies that can counteract immune evasion strategies and improve treatment outcomes for EC patients.

## Signaling pathways and cytokines

### Signaling pathways in the crosstalk between immune and tumor cells in EC

The intricate interplay between immune cells and tumor cells within the microenvironment of EC is orchestrated by an intricate network of signaling pathways. A profound understanding of these pathways is paramount in decoding the dynamics of cancer progression and formulating precise therapeutic strategies. The signaling pathway governed by interferons (IFNs) is central in regulating immune responses within the context of EC.^87^^,^^88^ Type I and type II IFNs, released by immune cells in response to the presence of tumor cells, serve as initiators of a cascade of events. These IFNs engage Janus kinases, which subsequently phosphorylate signal transducer and activator of transcription (STAT) proteins. These activated STAT proteins then translocate to the nucleus, where they assume control of the transcription of IFN-stimulated genes. Notably, the IFN-stimulated genes encompass elements such as major histocompatibility complex molecules, which play a pivotal role in promoting antigen presentation and the recognition of T cells.^89^^,^^90^

**Figure 4:**
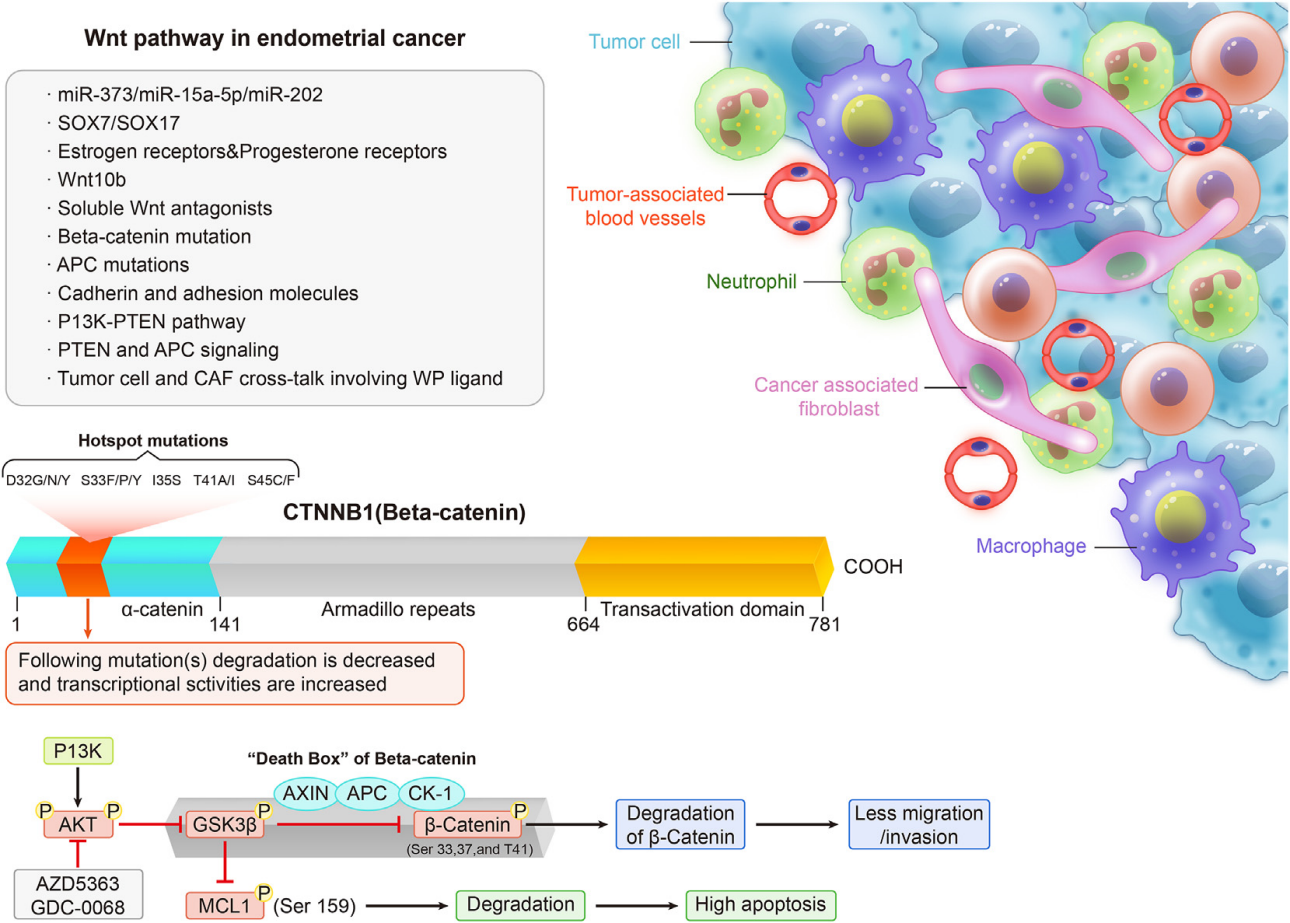
The Wnt signaling pathway has emerged pivotal in the context of endometrial carcinoma (EC). This signaling cascade has a multifaceted influence on the tumor microenvironment by orchestrating recruitment and activation of immune cells. Tumor-secreted Wnt ligands elicit responses from immune cells, promoting their migration and interaction with cancer cells. These intricate interactions highlight the significance of exploring the role of the Wnt pathway within EC. By understanding how Wnt signaling modulates immune responses, researchers can tailor therapeutic strategies and enhance anti-tumor immunity in this gynecological malignancy.

Toll-like receptors (TLRs), primarily expressed on immune cells, are instrumental in detecting pathogen-associated molecular patterns and damage-associated molecular patterns. TLR signaling is responsible for initiating inflammatory responses and activating nuclear factor-kappa B and IFN regulatory factors.^91^^,^^92^ In the unique microenvironment EC, TLR signaling serves to promote immune cell recruitment and activation. Notch signaling, a highly conserved pathway, is recognized for its influence on cell fate determination and the development of immune cells. In the context of EC, Notch signaling can modulate the phenotype and functionality of immune cells resident in the TME. The Wnt signaling pathway is indispensable for the recruitment and activation of immune cells within the confines of the TME^93^^,^^94^ (Fig. 4). Tumor cells release Wnt ligands, which hold the ability to attract immune cells, thereby facilitating their migration and fostering interactions with cancer cells.

A profound understanding of the interplay between these signaling pathways within the context of immune and tumor cell crosstalk is paramount in unraveling the complexities of EC. Targeting these pathways holds the promise of fostering innovative therapeutic strategies that amplify anti-tumor immunity and advance patient outcomes.

### Cytokines and chemokines in immune responses within the EC microenvironment

Cytokines and chemokines emerge as central mediators orchestrating immune responses within the unique milieu of the EC TME.^95^^,^^96^ These signaling molecules play a pivotal role in shaping the behavior of immune cells, influencing inflammation, and regulating the complex interplay between the tumor and the immune system. Several interleukins, such as IL-2, IL-10, and IL-12, have assumed prominence within the realm of EC. For instance, IL-2 is recognized for its role in up-regulating the proliferation and activation of T cells, thereby contributing to anti-tumor immunity. Conversely, IL-10 shows its immunosuppressive effects by inhibiting T-cell responses. Tumor necrosis factor-alpha, a pro-inflammatory cytokine, wields the capacity to instigate apoptosis in cancer cells and facilitate the recruitment of immune cells to the TME.^97^ It plays a dual role in cancer, promoting anti-tumor responses while simultaneously contributing to tumor-associated inflammation.

TGF-β stands out as a crucial immunosuppressive cytokine, capable of inhibiting the activation and function of T cells and nurturing the differentiation of regulatory T cells. C–C chemokine ligand 2 (CCL2) is responsible for recruiting myeloid cells, such as monocytes and macrophages, to the TME. This recruitment affects inflammation and immune responses in the realm of EC. Additionally, C-X-C motif chemokine ligand 12 (CXCL12) plays a crucial role in bringing immune cells, including T cells and dendritic cells, to the TME. The signaling associated with CXCL12 serves to influence immune cell infiltration and their interactions with cancer cells.

A comprehensive understanding of the roles of these cytokines and chemokines is necessary for unraveling the complexities of immune responses within the EC microenvironment. The pursuit of targeted therapeutic strategies holds the potential to modulate immune cell behavior, ultimately enhancing anti-tumor immunity within this gynecological malignancy.

### Literature evidence of the implication of pathways and molecules in EC regulation

Several studies have furnished compelling evidence regarding the multifaceted interactions between tumor-infiltrating immune cells, the intricate network of signaling pathways, and the crucial molecules within the TME, thereby advancing our comprehension of the disease and its prospects for therapeutic intervention. The association between elevated expression of PD-L1 and a more severe prognosis underpins the critical role of this immune checkpoint in immune evasion and disease progression. IFN-γ, serving as a key player in anti-tumor immunity, activates immune cells, especially cytotoxic T cells, thereby bolstering their ability to recognize and eliminate cancer cells.^98^^,^^99^

In the distinctive landscape of EC, TLR signaling plays a crucial role in recruiting and activating immune cells, which helps in fostering immune responses against cancer cells.^100^^,^^101^ These signaling molecules contribute significantly to immune cell infiltration and their interactions with cancer cells, consequently shaping the disease’s progression.^102^^,^^103^ The growing body of research highlights the complex nature of immune regulation in EC and emphasizes the important roles of specific pathways and molecules within this framework. This comprehensive understanding serves as the bedrock for the formulation of targeted therapeutic approaches aimed at enhancing anti-tumor immunity and the outcomes for patients with this gynecological cancer.

## Clinical implications and therapeutic strategies

### Clinical significance of elucidating molecular mechanisms in EC

The clinical importance of understanding the intricate molecular mechanisms governing interactions between tumor-infiltrating immune cells and EC cannot be overstated. Such knowledge advances our grasp of the ailment and holds substantial potential for refining patient care, therapeutic approaches, and overall clinical outcomes.^104^^,^^105^ A profound comprehension of the molecular intricacies inherent to EC equips us with the tools to formulate individualized therapeutic strategies. Medical practitioners can customize immunotherapies to align with each patient’s unique immune profile by discerning the specific immune evasion strategies deployed by the tumor. This precision medicine approach augments the probability of therapeutic success while minimizing unwarranted side effects.

The discovery of these molecular mechanisms paves the way for innovative immunotherapies by targeting key pathways and immune checkpoints, enhancing anti-tumor responses. Clinical trials of immune checkpoint inhibitors show promise for improving EC prognosis. Profiling molecular biomarkers helps in early detection and prognostication, and guiding precise therapeutic decisions, thus aiding in accurate disease staging and prognosis.

Understanding of molecular mechanisms is a pivotal gauge for assessing the response to treatment. The evaluation of transformations in the TME, the composition of immune cells, and immune evasion strategies during the therapy holds the potential to assess the efficacy of treatments. Real-time monitoring is indispensable for tailoring patient-specific therapeutic regimens and optimizing the outcomes. Grasping the molecular underpinnings of immune evasion clears the path for creating treatments pinpointed exclusively at the TME. This, in turn, minimizes off-target effects and mitigates treatment-related toxicity.

In summary, the clinical importance of unraveling the molecular intricacies underpinning interactions between the immune system and tumor cells in EC is profound. This knowledge informs personalized treatment strategies, propels advancements in immunotherapy, aids in early disease detection and prognostication, enables the ongoing assessment of treatment response, and ultimately elevates the standard of patient care. Research in this area offers hope for individuals afflicted with this cancer and paves the route to more efficacious and tailored therapeutic interventions.

### Targeting tumor-infiltrating immune cells in EC

Immunotherapies in the landscape of EC have focused on targeting tumor-infiltrating immune cells and have emerged as a promising frontier. These therapies harness the immune system of the patient to recognize and eliminate cancer cells. Numerous clinical trials have evaluated the efficacy of such therapeutic modalities, providing invaluable insights into their impact on disease management.

Innovative therapeutic modalities such as chimeric antigen receptor-T cell therapy and tumor-infiltrating lymphocyte therapy, where a patient’s T cells are carefully modified to enhance their anti-tumor functionality, are currently under exhaustive study in the context of EC.^106^^,^^107^

Additionally, therapeutic vaccines targeting tumor-specific antigens have displayed substantial potential within the crucible of clinical trials. The VERSATILE trial evaluated a personalized neoantigen vaccine in patients with mismatch repair-deficient EC, yielding encouraging results eliciting anti-tumor immune responses. Furthermore, clinical trials frequently evaluate combination therapies that augment the efficacy of immune-based treatments. Concomitantly, targeted therapies, such as the combination of lenvatinib and pembrolizumab, have shown promise in patients afflicted with advanced EC. The KEYNOTE-146 trial witnessed notable responses achieved with this therapeutic amalgamation in select patient subsets.^108^

The advancement of clinical trials highlights the potential of immunotherapies and combination strategies in managing EC. While certain patients have reaped substantial benefits from these treatments, the continuum of research endeavors further finetunes the process of patient selection and therapeutic regimens, ultimately optimizing treatment outcomes. These advancements breathe fresh hope into the lives of patients suffering from this gynecological cancer, underscoring the transformative role played by immunotherapies within the context of cancer management.

## Future directions and unresolved questions

Despite significant progress in understanding the immune microenvironment in EC, a comprehensive exploration of the underlying mechanisms of immunosuppression is urgently needed. Studying immune checkpoints, cytokines, and regulatory T cells is key to developing effective strategies against immune evasion. As identification of reliable biomarkers for predicting responses to immunotherapies is crucial, research should focus on uncovering molecular signatures and immune cell profiles to guide patient selection for immunotherapies, and enhance efficacy while minimizing side effects.

The variegated landscape of tumor-infiltrating immune cells in the context of EC has been inadequately explored. Future research should focus more deeply on understanding the different types and functions of immune cells in the TME. Discerning their distinct roles and intricate interactions lends invaluable insights for fine-tuning therapeutic strategies. The formidable challenge of resistance to immune checkpoint inhibitors necessitates rigorous investigation. The underlying mechanisms, including secondary immune checkpoints and adaptive immune responses, should be subjected to detailed scrutiny to unshackle novel pathways for transcending treatment limitations. Exploring the combination of immunotherapies with other treatments, like targeted therapies or radiation therapy, is a promising direction for future research. Research endeavors should explore optimal combinations and their potential to enhance response rates and elevate patient care.

EC’s genetic and phenotypic diversity presents a significant challenge for devising targeted therapies. Variations in tumor cells' responsiveness to immunotherapies complicate patient stratification. Another major obstacle is the lack of representative preclinical models accurately reflecting EC and its immune microenvironment. Relevant models need to be developed to translate research findings into clinical applications. Selecting patients likely to respond favorably to immunotherapies is a burdensome process. To this end, refining stratification criteria and identifying novel biomarkers are essential for effectively guiding treatment decisions.

Timely assessment and prediction of responses to immunotherapies offer significant potential for enhancement. Improved imaging techniques and advanced essential immune monitoring tools are crucial for optimizing treatment regimens. While combination therapies hold promise, identifying optimal combinations and sequences of interventions requires further assessment; it is essential to balance therapeutic efficacy and potential side effects.

In summary, investigating EC and the interaction between tumor-infiltrating immune cells is a dynamic field with various avenues for future research. Fundamentally, knowledge gaps, such as understanding immunosuppression and predicting treatment responses, need to be bridged and challenges like tumor heterogeneity and patient selection must be addressed. Collaboration among researchers, clinicians, and industry partners is paramount for advancing this critical realm of cancer research.

## Conclusion and discussion

The complex and ever-evolving interplay between EC and infiltrating immune cells is a dynamic arena that has attracted the collective attention of researchers as well as clinicians. This review offers a nuanced perspective, elucidating key findings and highlighting the areas for investigation. These insights highlight the profound implications that extend to the diagnosis and therapeutics for this formidable gynecological malignancy. This review attempts to comprehend the molecular mechanisms driving immune evasion by EC cells. The PD-1/PD-L1 pathway has assumed a central role in the orchestration of immune regulation. Clinical trials have shown the potential of immune checkpoint inhibitors, notably pembrolizumab and nivolumab, in augmenting patient outcomes. Additionally, IFN signaling, TLR signaling, cytokines, and chemokines have garnered recognition for their substantial roles in sculpting the immune response within the microenvironment.

This discussion emphasizes the critical need for customizing treatment approaches in alignment with the distinctive immunological profile of each patient. Notably, a significant gap in knowledge exists in the area of biomarkers that can predict the response to immunotherapies. Identifying such markers augments treatment efficacy and simultaneously acts as a safeguard against the onset of deleterious side effects. The sphere of tumor-infiltrating immune cell heterogeneity beckons as an enigmatic avenue for prospective research. Exploring the range of phenotypic and functional diversity of immune cell populations within the microenvironment may offer valuable insights that could be used to tailor treatment strategies. Investigating the mechanisms behind resistance to immune checkpoint therapies and combining different treatment approaches open up new possibilities for innovative solutions, potentially improving response rates and enhancing overall patient care.

Nevertheless, it is crucial to acknowledge challenges and limitations. The complexity of tumor diversity and the challenges in selecting the right patients highlight the need for improved preclinical models and predictive tools. The field confronts the formidable challenge of sculpting an optimal symphony of combination therapies that balance therapeutic efficacy with the mitigation of potential side effects.

In summation, this research is not just academically important but also has a significant impact on improving clinical care for patients with EC. The advent of immunotherapies, coupled with the strides made in illuminating the complex immune–tumor interactions within the microenvironment, has already borne witness to a transformation in patient outcomes. Yet, this review strongly emphasizes the need for an enduring commitment to ongoing research, the evolution of innovative therapeutic strategies, and the meticulous refinement of predictive biomarkers, all with the common aim of further elevating the efficacy of EC treatment.

The progress in this field relies on strong teamwork among researchers, doctors, and industry partners. The voyage towards improved treatment options for patients grappling with EC remains an ongoing saga, and the progress made so far suggests the possibility of groundbreaking discoveries that could greatly improve the outlook and quality of life for those affected by EC.

## CRediT authorship contribution statement

**Silu Ding:** Data curation, Investigation, Writing – original draft. **Yingying Hao:** Data curation, Investigation, Writing – original draft. **Yue Qi:** Data curation, Investigation, Writing – original draft. **Heng Wei:** Conceptualization, Writing – review & editing. **Jin Zhang:** Conceptualization, Writing – review & editing. **Hui Li:** Conceptualization, Writing – review & editing.

## Conflict of interests

The authors have no competing interests to declare.
